# A Possible Association Between Rituximab and the Subsequent Development of Guillain–Barré Syndrome: A Case Report

**DOI:** 10.3390/reports8030119

**Published:** 2025-07-23

**Authors:** Lilian Chen, Stephen Lee Yu, Nolan Holley, Salahuddin Safi

**Affiliations:** 1 Department of Internal Medicine, West Virginia University, Morgantown, WV 26506, USA; nolan.holley@hsc.wvu.edu; 2 Department of Medical Oncology, West Virginia University, Morgantown, WV 26506, USA; stephen.yu@hsc.wvu.edu (S.L.Y.); salahuddin.safi@hsc.wvu.edu (S.S.)

**Keywords:** hematology, neurology, peripheral nerve disease, rituximab, Guillain–Barré syndrome

## Abstract

**Background and Clinical Significance**: Hematologic malignancies, including diffuse large B-cell lymphoma (DLBCL), have been associated with the development of Guillain–Barré syndrome (GBS). Specifically, treatment with the immunomodulator rituximab, which is used in the backbone of DLBCL treatment, has increasingly been used in this patient population. **Case Presentation**: We present the case of a man in his 60s with DLBCL who presented to the hospital with the progressive weakness of the bilateral upper and lower extremities within 6 weeks of the completion of treatment including rituximab. The temporal relationship between the completion of rituximab and subsequent polyradiculoneuropathy, as well as a favorable response to intravenous immunoglobulin (IVIG), affirmed the diagnosis of treatment-induced GBS. **Conclusions**: The increased use of rituximab as part of a standard treatment regimen for hematologic malignancies demonstrates the need for an awareness of a possible association between rituximab and the subsequent paradoxical development of GBS, which will allow for expeditious evaluation for better patient outcomes.

## 1. Introduction and Clinical Significance

Diffuse large B-cell lymphoma (DLBCL) is the most common type of non-Hodgkin lymphoma (NHL) [1]. DLBCL is characterized by the rapid and aggressive development of expansive lymphadenopathy and systemic symptoms such as night sweats, fever, and weight loss. Prompt diagnosis and initiation of treatment are critical for this patient population. Remarkable progress has been made over the past several decades with regard to the prognosis for patients with DLBCL. The mortality rate for DLBCL in 2020 was 1.6 per 100,000 persons, which has improved from the rate of 2.8 per 100,000 persons seen in 1992 [2]. This improvement is largely attributable to advancements in the management of DLBCL during this period. In 1997, rituximab earned FDA approval for the treatment of B-cell NHL and was combined with cyclophosphamide, doxorubicin, vincristine, and prednisone to form the current standard-of-care treatment for DLBCL, commonly referred to as R-CHOP [3].

Guillain–Barré syndrome (GBS) typically presents as rapidly progressing ascending paralysis, beginning symmetrically in the lower limbs and progressing superiorly [4]. There have been a few reported cases that have described possible relationships between DLBCL, R-CHOP, and GBS [5,6,7]. Even in the setting of these few reported cases, it remains difficult to differentiate whether the development of GBS is related to R-CHOP treatment or a paraneoplastic syndrome secondary to the disease process itself. Several prior reports have described GBS developing after rituximab. For example, Terenghi et al. reported on a patient with follicular lymphoma who developed GBS two weeks after rituximab therapy [5]. Additionally, Carmona et al. described a similar case in which a patient with rheumatoid arthritis who was treated with rituximab developed GBS one month later [6]. More recently, a patient with DLBCL developed GBS just three weeks after rituximab, necessitating plasmapheresis for eventual recovery [7]. To further contribute to the current literature, we present the case of a male in his 60s with DLBCL who developed GBS three months after completing R-CHOP therapy.

## 2. Case Presentation

### 2.1. Case Presentation

A physically active man in his 60s with a history of DLBCL and a chronic neurogenic bladder, for which he has self-catheterized daily for many years, presented to the hospital with concerns of generalized fatigue, weakness, and dyspnea. He completed six cycles of R-CHOP four months prior to his presentation. One month prior to his presentation to the hospital, he reported a left-sided facial droop and was diagnosed with Bell’s palsy. He was prescribed a 10-day course of prednisone, which led to the complete resolution of his facial droop. As the weeks passed, he started to experience intermittent numbness in his hands and constant numbness and tingling from the level of his umbilicus and radiating down his lower extremities. He visited his local emergency department (ED) for his symptoms and underwent computed tomography imaging of his head, abdomen, and pelvis, which only revealed a small amount of possible blood in the bladder. Work-up during his ED visit revealed a possible urinary tract infection, for which he completed a course of antibiotics and was discharged to follow-up with his family physician. He reported that he was extremely weak and unable to walk upright, which is very unusual for him as he continued to work a physically demanding job during his chemotherapy treatments. He denied any exposures to pesticides, any recent fevers, or recent trauma. On the day of the hospital presentation, he reported having an inability to even step up to get into a vehicle and had to crawl on his hands and knees into the house to be brought to the hospital by a family member.

A physical exam revealed absent reflexes in the bilateral upper and lower extremities as well as moderate proximal and distal weakness (3/5 in strength bilaterally). He was also noted to have sensory loss to light touch and a pinprick sensation from the umbilicus to the distal toes, as well as vibration and proprioception loss on the lower extremities. There were no apparent cranial nerve deficits, and no Babinski signs were observed. Given his recent completion of chemotherapy and a concern for chemotherapy-induced disease process, he was admitted to the hematology service for further evaluation and management.

### 2.2. Investigations

The magnetic resonance imaging (MRI) of the brain and thoracic, lumbar, and sacral spine revealed an indeterminate enhancement in the posterior superior T11 vertebral body. Upon radiologist review, this finding was considered suggestive of either malignancy or hemangioma, with follow-up imaging recommended for further elucidation. Inflammatory markers revealed a white blood cell count of 6.1 × 10^3^/μL (ref.: 4.5–11 × 10^3^/μL), C-reactive protein levels of 8.6 mg/L (ref.: <1 mg/L), and an erythrocyte sedimentation rate of 36 mm/h (ref.: 0–15 mm/h). Infectious work-up, including urinalysis and urine culture, blood cultures, and a respiratory viral panel, were negative, and there was no evidence of metabolic derangements.

Neurology was consulted due to concerns about a polyradiculoneuropathic process. A lumbar puncture (LP) revealed elevated protein levels (85 mg/dL; ref.: 14–45 mg/dL), decreased glucose levels (55 mg/dL; ref.: 60–80 mg/dL), lymphocytic predominance (96% lymphocytes, 4% monocytes), and cytopathology negative for malignant cells. Additional cerebral spinal fluid tests for bacterial and fungal cultures, Epstein–Barr virus, JC polyomavirus, Borrelia, syphilis, cytomegalovirus, cryptococcus, enterovirus, and West Nile virus were negative. BK virus was not tested for. Flow cytometry demonstrated essentially no B-cells (<0.1%) on pathologist review. Notably, a multiple sclerosis panel revealed one well-defined oligoclonal band and the elevation of immunoglobulin G (IgG) at 1990 mg/dL. Electromyography (EMG) demonstrated active denervation changes in the left lumbosacral paraspinals suggestive of left lumbosacral radiculopathy, but it was not possible to exclude a superimposed generalized neuropathy. Conduction blocks and temporal dispersion were not seen. Although it is unusual for EMG findings to be normal a few weeks into a patient’s disease course, a negative EMG does not definitively rule out a diagnosis of GBS if the clinical course is suggestive of it and other investigations support the diagnosis [8]. In this case, although the LP and EMG findings were equivocal for a diagnosis of GBS, the extensive negative work-up, combined with the physical exam findings of areflexia and progressive weakness originating in the lower extremities and later involving the upper extremities, was highly compelling for a diagnosis for GBS. This warranted consideration for prompt empiric treatment.

### 2.3. Differential Diagnosis

Differential diagnoses also included, though were not limited to, electrolyte disturbances, acute cord abnormalities, neurolymphomatosis, and chemotherapy-induced neuropathy. Laboratory markers were not suggestive of acute derangements. Given the sensory level at the umbilicus as well as enhancement at T11 on imaging, we were unable to completely rule out a symptomatic cord lesion; however this was lower on the differential as the patient’s symptoms were polyradiculoneuropathic in nature and involved the upper extremities. With the history of cancer, neurolymphomatosis was considered. However, the cytopathology was negative for malignant cells, and the patient did not present with characteristic pain or cranial nerve involvement. While the oligoclonal band and elevated IgG could be suggestive of a lymphoma process, it is a nonspecific finding. Furthermore, the absence of B-cells on flow cytometry effectively ruled out CNS lymphoma. Finally, chemotherapy-induced neuropathy was ruled out given the timeline of symptom development as this diagnosis would have been supported with a more chronic course and more distal symptoms.

According to the Brighton criteria for GBS diagnosis, our patient met level 1 diagnostic certainty, the highest level of diagnostic confidence. Specifically, he demonstrated bilateral and flaccid weakness of the lower extremities, decreased or absent deep tendon reflexes in the lower extremities, progressive worsening over 10–14 days, CSF protein elevation with low cell count, and the results of nerve conduction studies were consistent with acute inflammatory demyelinating polyneuropathy. While antiganglioside antibody testing was not performed, as it is not required for diagnosis under the Brighton criteria and would not have altered acute management, it may have provided additional information regarding specific GBS variations. Collectively, these findings support a robust diagnosis of GBS in this patient despite some limitations.

### 2.4. Treatment

The patient was treated with intravenous immunoglobulin (IVIG) at 400 mg/kg for five days. He also underwent physical therapy (PT) and occupational therapy (OT) frequently while he was admitted in the hospital.

### 2.5. Outcome and Follow-Up

Throughout the patient’s admission, he had gradual improvement in strength while receiving his IVIG administration in conjunction with PT and OT. On initial neurological examination at the time of presentation, he demonstrated Medical Research Council grade 3/5 strength in bilateral hip flexors and knee extensors, 2/5 strength in bilateral ankle dorsiflexors and plantarflexors, and absent reflexes throughout the lower extremities. His GBS disability score was grade 4, as he was bedbound. At the time of hospital discharge following the completion of IVIG, strength improved to 4+/5 in hip flexors and knee extensors 4−/5 in ankle dorsiflexors and plantarflexors, and the partial return of patellar reflexes (1+) was seen. His GBS Disability Scale score improved to grade 2, as he was able to walk 10 or more meters with assistance. During his one-month outpatient follow-up after discharge, his physical exam continued to demonstrate hyporeflexia as well as proximal and distal weakness to the extremities; however, the patient self-reported subjective signs of improvement to his weakness and was regaining functional status. Since his weakness had not progressed since discharge, there was no indication for the repeat administration of IVIG, and the patient was instructed to continue outpatient PT. Five months after discharge, the patient continued to have improved strength in his lower extremities and no longer required any assistance devices on ambulation, and he is back to being able to perform physically demanding work.

## 3. Discussion

GBS is an acute immune-mediated polyradiculoneuropathy that occurs due to the development of autoantibodies against antigens specific to neurons. Clinically, this typically manifests as an ascending flaccid paresis that commonly initiates in the distal lower extremities. Diagnosis is made based on a combination of clinical features, cerebrospinal fluid analysis, and electrodiagnostic studies. When diagnosing GBS, it is critical to rule out other differential diagnoses including alternative peripheral polyradiculoneuropathies, alternative myopathies, central nervous system pathology, metabolic diseases, infectious diseases, electrolyte disorders, and vitamin deficiencies [9]. While GBS most commonly occurs as a sequelae of recent respiratory or gastrointestinal infection due to molecular mimicry, it has been an infrequently documented adverse effect related to the immunotherapy treatment of malignancies [5,6,7].

In recent years, there have been an increasing number of studies investigating a possible association between cancer and the development of GBS. A recent population-based study in Denmark has shown that patients with a recent diagnosis of cancer within the past 6 months prior to the development of GBS had 3.6 times the risk of the general public, with those with a hematologic malignancy demonstrating 7 times the risk [10]. One proposed mechanism for the development of polyradiculoneuropathy in association with hematologic malignancy, particularly B-cell lymphomas, is through malignant cells infiltrating the nerve parenchyma, also known as neurolymphomatosis. There are varied clinical manifestations of neurolymphomatosis, often asymmetric mononeuropathies; however, there have been rare cases of the development of a symmetric polyradiculoneuropathy like GBS [11].

**Figure 1:**
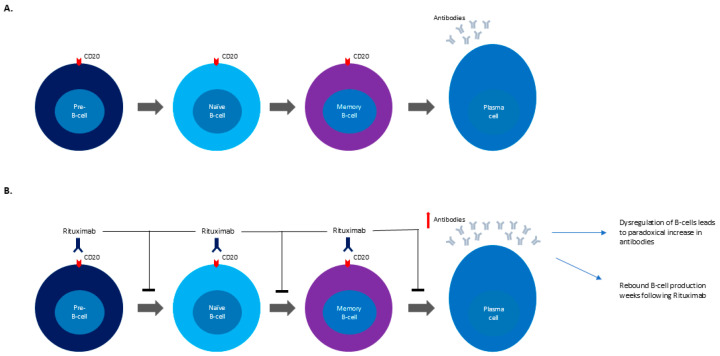
(**A**) Mechanism for plasma cell development (**B**) Mechanism for GBS development following rituximab treatment.

While there have been increasing studies investigating the association between hematologic malignancies and the development of GBS, the possible relationship between immunotherapy and GBS is infrequently documented and less well understood. Few case reports have documented the development of GBS as a result of rituximab based on the temporal relationship between treatment with rituximab and the development of GBS [5,6,7]. This relationship is controversial given rituximab’s utility in treating autoimmune conditions such as rheumatoid arthritis, granulomatosis with polyangiitis, and other cases of antineutrophil cytoplasmic antibody-associated vasculitis. This suggests that rituximab has a role in the treatment of GBS rather than as a cause of GBS [7]. Specifically, rituximab’s role in autoimmune diseases is to target CD20-positive B-cells, which are a required intermediary in the creation of plasma cells [12]. While some authors postulate that B-cell dysregulation may paradoxically increase neuronal antibodies, resulting in the development of GBS, there has been limited research into the exact mechanism behind this relationship (Figure 1A,B) [7,13]. It should also be noted that vincristine, another component of R-CHOP, has been noted to cause sensory–motor axonal neuropathies in increasing doses; however these cases often include autonomic dysfunction or cranial nerve involvement in addition to the distal to proximally advancing weakness [14]. These symptoms were not present in our patient’s case which made vincristine-induced neuropathy less likely in our differential.

Our patient’s case was not a typical presentation of GBS. Rather than the typical presentation of ascending paralysis, he had demonstrated descending paralysis from the umbilicus, descending with further sensory losses to the distal trunk and lower extremities. He had a very extensive infectious work-up completed in both blood and cerebrospinal fluids that were all negative for a causative organism. Furthermore, his LP and EMG findings were equivocal for a diagnosis of GBS. However, given the temporal relationship within a few months between the completion of combination chemotherapy, including rituximab, for the patient’s DLBCL and the subsequent progressive development of polyradiculoneuropathy, the most compelling diagnosis was GBS, with rituximab as the most likely etiology. Of note, rituximab’s clearance in DLBCL treatment for men averages at 24.7 days, and our patient’s symptoms developed four months prior to his presentation [15]. This temporal gap between the completion of treatment and symptom presentation was unusual; however in the absence of other provoking factors for the development of his symptoms, rituximab-induced neuropathy remained the most likely explanation of our patient’s diagnosis.

Our patient’s clinical response to IVIG administration also favored this diagnosis. Additionally, while a positive antiganglioside antibody finding would have further supported the diagnosis of GBS, it was not obtained during initial work-up. Given the patient’s improvement with IVIG treatment, further testing was deemed unnecessary as it would not have altered management. Ultimately, the patient met European Academy of Neurology/Peripheral Nerve Society’s required criteria of the progressive weakness of the upper and lower extremities and areflexia of the affected limbs all within a four-week time course, supporting the diagnosis of GBS in this case [8]. The differential diagnosis for our patient’s acute flaccid paralysis also included neurolymphomatosis, paraneoplastic syndromes, and vincristine-induced neurotoxicity. Neurolymphomatosis was considered given his underlying DLBCL; however, it was deemed not likely due to the absence of cranial nerve involvement or neuropathic pain, negative CSF cytology, and a lack of radiographic evidence. Paraneoplastic syndromes were considered but unlikely given the absence of onconeural antibodies or systemic paraneoplastic features. Vincristine-induced neurotoxicity was investigated. The patient was given a cumulative dose of 12 mg (2 mg per cycle × 6 cycles), which is within standard dosing for R-CHOP. Vincristine-induced neurotoxicity typically presents as a length-dependent sensory neuropathy with autonomic manifestations, which was not present in our patient. Collectively, these considerations taken together with his clinical presentation and objective findings supported a diagnosis of GBS rather than alternative etiologies.

## 4. Conclusions

Guillain–Barré syndrome is an immune-mediated polyradiculoneuropathy that requires prompt recognition and treatment to reduce morbidity. While rituximab-based regimens such as R-CHOP are generally well-tolerated from a neurological perspective, rare complications such as GBS have been reported. This case describes GBS following R-CHOP for DLBCL, with improvement in neurological symptoms after IVIG. This case further contributes to the growing evidence suggesting a possible association between R-CHOP and GBS and underscores the importance of maintaining a broad differential diagnosis in patients presenting with acute flaccid paralysis.

## Data Availability

The original contributions presented in this study are included in the article. Further inquiries can be directed to the corresponding author.
